# From Proteomics to the Analysis of Single Protein Molecules

**DOI:** 10.3390/ijms251910308

**Published:** 2024-09-25

**Authors:** Elena A. Ponomarenko, Yuri D. Ivanov, Anastasia A. Valueva, Tatyana O. Pleshakova, Victor G. Zgoda, Nikita E. Vavilov, Ekaterina V. Ilgisonis, Andrey V. Lisitsa, Alexander I. Archakov

**Affiliations:** Institute of Biomedical Chemistry, 10, Pogodinskaya St., 119121 Moscow, Russia

**Keywords:** single biomacromolecule, molecular counters, sensitivity in bioanalysis, protein detection, atomic force microscopy, proteomics, mass spectrometry, limit of detection

## Abstract

Limit of detection (LoD) is a term that is used to characterize the sensitivity of an analytical method. The existing limitation of the sensitivity of analysis using modern mass spectrometry methods has been experimentally shown to be a limiting factor in the application of proteomic technologies in medicine. This article proposes a concept of a new technology that will set a new vector of development in the development of systems for solving problems of medical diagnostics and deals with theoretical and practical aspects of creating a new technology for the detection of single biomacromolecules (in particular, proteins) in biological samples. Such technology should be based on the principle of signal registration similar to that used in a Geiger counter (also known as a Geiger–Müller counter or G-M counter), a device that automatically counts the number of ionizing particles that hit it. This counter is free from probabilistic components; it registers a signal if there is at least one target molecule in the analysis chamber. Predictive medical diagnostics require technology based on methods where sensitivity allows for the detection of single marker molecules in a biological sample volume of 1–10 µL, the smallest volume of biomaterial used in laboratory diagnostics. Creation of a detector with a sensitivity of 10^−18^ M would allow for the detection of one molecule in 1 µL of the sample, which fundamentally makes this approach analogous to a G-M counter for solutions. To date, bioanalytical methods are limited to a sensitivity of 10^−12^ M (which is approximately 1 million molecules per 1 μL), which is insufficient to capture the early stages of pathological processes.

## 1. Introduction

Since the early 2000s, post-genomic research methods have started to advance rapidly [1,2]. Upon that time, substantial time and material resources have been invested in the development of protein analysis methods [3,4,5,6,7,8,9]. In 2001, a large-scale project in the field of living systems research was initiated, the Human Proteome Project [10]. It was expected that its results would lead to a revolution in medical diagnostics by registering new biological markers—proteins associated with the occurrence or development of pathological conditions [11]. This has not been the outcome; since 2001, the list of FDA-approved marker proteins has included only 17 items used in diagnostics [12].

For medical diagnostics, it is important to use biological samples in liquid form, since collection of this type of samples is a minimally invasive and convenient procedure compared to tissue biopsy [13,14,15,16,17]. Liquid biopsy has several advantages in addition to being minimally invasive and easy to obtain. It is virtually painless and accompanied by minimal risk, and allows for extrapolation of large data using multiomics technologies and monitoring of physiologic and pathologic conditions over time based on molecular composition [18].

Modern analytical approaches should be focused on determining the number of target molecules in dilute biological samples (solutions), and must enable the precise quantification of biomolecules in dilute solutions.

The basis of the new analytical approach can be equipment that allows for registering the signal from each biomolecule, i.e., molecular detectors or molecular biological counters. Currently, modern mass spectrometric detectors can detect the presence of a compound if it is present in an amount of at least 1000 molecules per 1 µL [9].

Molecular counters in biology can be compared to G-M counters in physics [19], which are counting molecules that assess the level of their radioactivity. The counters record the presence of each ionized molecule that enters the analysis area, i.e., the fact that a particle passes through the detector chamber is recorded. The detector signal is caused by the presence of a radioactive particle and the volume of the chamber is not a factor in determining the sensitivity of the measurement system.

Developing the concept of creating an analog of a G-M counter for solving problems in biology and medicine requires preliminary calculations. As a result of the calculations, it is necessary to (a) estimate the number of molecules in a sample of biological material required for the determination of the existing analytical method with a given sensitivity; (b) establish the potential of using experimental and bioinformatic technologies to determine the complete proteome of complex biological samples.

The aim of the publication is to highlight the need for a new bioanalysis technology. The shortcomings of current methods for protein detection are demonstrated through calculations and examples of experimental results. Using calculations, the sensitivity of the method that should be created to detect all proteins in a biological sample is demonstrated. The new method should provide registration of signals from each molecule of a protein in the analyzed volume. The calculated sensitivity of the assay is 10^−18^ M, which corresponds to one protein molecule in 1 µL. In our opinion, only in this case a complete inventory of all proteins in the organism can be carried out, which will allow us to find among them biomarkers of disease/health status.

## 2. Estimating the Number of Molecules in a Sample of Biological Material

The task is determined by the nature of the biological samples to be analyzed, which are predominantly liquids (blood, urine, saliva, etc.). They are solutions that are multi-component systems of biomolecules and salts in water. The term molar concentration is used to express the content of a substance in a solution (moles of a substance in a liter, denoted by M [20]). For the detection of biomolecules, this notion, which is defined as the number of biomolecules per unit volume of solution, is often used to analyze biological solutions.

At the same time, it is shown [21] that molar concentration characterizes not only the number of biomolecules, but also the space (distance) between them: the higher the concentration, the smaller the space between molecules in the solution. According to these calculations, the distance between biomolecules in a 1 M solution is 1.18 nm, and in a 1 nM solution, it is 1180 nm. Decreasing the concentration by nine orders of magnitude leads to an increase in the distance between molecules by three orders of magnitude [21]. Spacing is a defining parameter in the study of the function of biomolecules; intermolecular interaction is possible if the distance between molecules is no more than 2–3 radii of these molecules, i.e., a few nanometers [22].

Regularities that are obtained for pure substances may not always be correctly applied to the description of complex biological material. For example, assuming that blood is a solution of proteins, it is necessary to take into account the role of albumin, the major blood protein, whose concentration reaches 10^−4^ M.

Let the concentration of 1 M be considered as a limiting case, reflecting the maximum possible number of biomolecules in solution and, accordingly, the minimum space between them: 1 M solution contains ~10^24^ molecules in a liter. According to calculations, the distance between these molecules is on average 1 nm [21]. This situation would be impossible for biological systems, since they contain a large spectrum of different types of biomolecules present in much smaller concentrations.

Another limiting case is the situation where a minimal number of biomolecules are present in a solution. This situation corresponds to a solution concentration of ~10^−24^ M, i.e., one molecule in one liter. The existence of such a system is quite possible: at the earliest stage of a biochemical (including pathological) process, only a single biomolecule appears in the organism. Such single biological markers cannot currently be detected due to the limitations of sensitivity of analytical methods, as millions or thousands of molecules are needed for a device to register the corresponding signal.

There is a need for an analytic method that detects these kinds of biomolecules at ultra-low concentrations, justified by the importance of early and predictive diagnoses. Calculations show that a tumor with a diameter of 1 mm (smaller than what can be registered by routine clinical methods) releases biomarkers into the blood at concentrations of 10^−15^ M (10^19^ molecules in 1 L of blood) [23], which corresponds to approximately 10^3^ marker molecules in 1 μL of blood, three orders of magnitude below the best current diagnostic technologies that detect compounds at a concentration of at least 10^−12^ M.

For predictive diagnostics, it is necessary to have analytical methods that detect single biomarker molecules in a volume of 1–10 µL (the smallest volume of biomaterial used in laboratory diagnostics). Depending on the concentration of the analyte, this volume may contain different numbers of molecules (see Table 1).

Let us assume that the sensitivity of the method is 10^−12^ M or 10^−15^ M, which corresponds to 10^6^ and 10^3^ biomolecules in 1 µL. As can be seen from the calculations, even at a concentration of 10^−15^ M, thousands of molecules are present in the solution. The analyte concentration of 10^−15^ M is inaccessible for detection by most modern analytical methods, since the accumulation of the minimum number of molecules necessary to trigger the detector requires increasing the volume of analyzed solutions to milliliters.

Thus, the creation of a detector with a sensitivity of 10^−18^ M would make it possible to detect one molecule in 1 µL of sample, which fundamentally makes this approach analogous to a G-M counter for solutions. To date, the methods are limited to a sensitivity of 10^−12^ M (which is approximately 1 million molecules per 1 μL), which is insufficient to register the early stages of pathological processes.

It is reasonable to have a minimum volume analysis of 1 µL and a maximum volume of 100 µL, since the ultimate goal is to develop medical test systems focused on screening studies when a small volume of biological sample is taken. The best sensitivity of a single molecule detector will be 10^−18^ M at a sample volume of 1 µL, and 10^−20^ M at 100 µL. A sample volume parameter in the counter device becomes crucial; if this volume is 1 µL, a solution with an analyte concentration of 10^−18^ M cannot be analyzed, as the probability of detecting a biomolecule in the sample becomes 0.01% and decreases tenfold when the concentration decreases by an order of magnitude.

**Figure 1:**
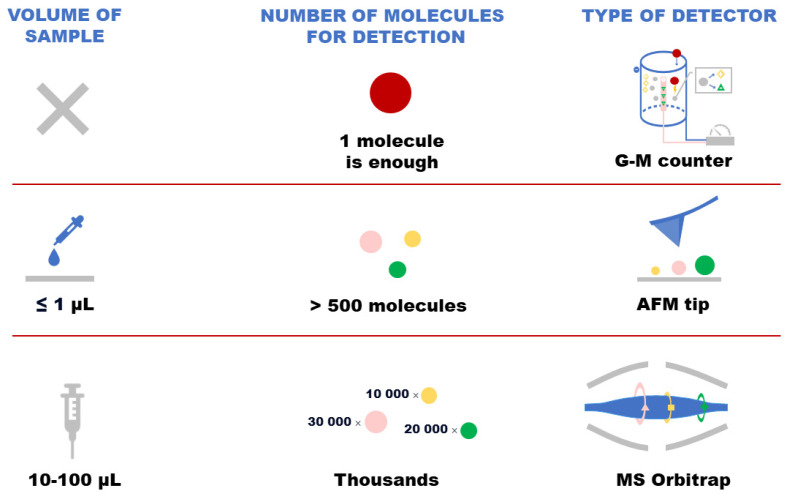
Demonstration of basics of G-M counter, AFM and mass spectrometric detectors. Top panel—G-M counter; middle panel—AFM; bottom panel—MS analysis.

It depends on the recording method underlying the single molecule detector whether the volume can be increased to 100 µL, but its sensitivity should be at least 10^−18^ M. Figure 1 shows the basics of G-M counter, AFM and mass spectrometric detectors. Top panel—G-M counter; middle panel—AFM; bottom panel—MS analysis.

Figure 1 (top panel) shows a schematic picture of a G-M counter. The G-M counter is a charged capacitor, the area between the electrodes of which is filled with gas, whose principle of operation is based on the ionization phenomenon. When an ionizing particle enters the chamber, it causes ionization of gas molecules to electrons and ions. The electrons are accelerated in the powerful electric field inside the device and begin to knock out other electrons. This happens until an electric current begins to flow between the cathode and anode. When the current begins to flow between the electrodes of the capacitor, this is called breakdown. The resulting voltage can be recorded and the radioactive background quantified. A single particle causes an avalanche of ionization, which makes it possible to record its presence in the counter chamber [25,26].

For example, when using atomic force microscopy (AFM) (Figure 1, middle panel), it is important to locate the detectable molecules on the surface, which is further scanned and counted by the number of molecules [27]. If a volume of 1 µL is applied on the surface, the liquid (depending on the hydrophilicity or hydrophobicity of the substrate) will occupy 100–1000 square microns. Such an area can be scanned in a reasonable amount of time (dozens of hours). If 100 µL is applied to the surface, molecules will have to be scanned on the surface with an area hundreds of times larger. Consequently, this would require substantial time resources, which is absolutely unacceptable for a medical diagnostic system. The theoretical limit of sensitivity of the AFM-based method is one molecule, but practically, values of 10^−18^ M can be achieved provided that the analyte volume is 1 µL and AFM is used instead of a mass spectrometric detector, despite the limitations in the scanning time of large surfaces.

The principle of the AFM operation is based on the registration of force interaction between the surface of the sample under study and the probe. The probe is a nanoscale needle at the end of an elastic arm (cantilever). The appearance of changes in topography (elevation, depression) under the needle leads to a change in the force acting on the probe, hence to a change in the amount of cantilever bending. By registering the bend value, it is possible to draw a conclusion about the surface relief features [28]. The AFM method can be used to visualize proteins and determine their heights. The protein molecule immobilized on the surface is elevated; the height can be determined as 0.1 nm with high accuracy. However, it is not possible to identify the protein by AFM. The result of AFM analysis is interpreted as the appearance of objects with a height greater than 1 nm, in an amount greater than the noise level (500 objects in an area of 400 µm^2^ [29]). The height range from 1 to 7 nm corresponds to the height of proteins immobilized on the surface. The method of biospecific recognition, which is widely used, for example, in enzyme-linked immunosorbent assay analysis (ELISA), can be applied for diagnostic purposes. Details of biospecific AFM analysis are described in [27]. In brief, a molecular probe (aptamer or antibody) is covalently immobilized on the atomically aligned surface of a substrate. AFM scanning of the surface reveals the original surface topography. Next, the substrate is incubated in a diluted biological sample (plasma or blood serum); during incubation, complexes of the target protein with the molecular probe are formed on the surface due to biospecific interactions. Subsequent AFM scanning registers these complexes on the basis of the appearance of objects with higher height on the surface topography compared to the original topography. Since the size of the AFM probe is comparable to the size of the target proteins and their complexes, unprecedented sensitivity of signal registration from each target protein is achieved. The main problem that exists in biospecific AFM analysis is the dissociation of molecular probe/protein complexes, which leads to a decrease in their amount on the AFM chip, reducing the sensitivity of the analysis. Another problem is the long analysis time. As shown in the demonstration in Figure 1 (middle panel), each protein molecule can be registered by AFM on the surface, but for reliable results, their number should exceed the number of non-specific objects (500 pieces).

Now consider another method of registration—the mass spectrometric detector. The Orbitrap mass detector shown in Figure 1 (bottom panel) allows for measuring the mass-charge ratio of ions of investigated substances with an accuracy of less than 1 ppm. Having outstanding resolution characteristics (ms characteristic, which allows for us to distinguish the peak of one *m*/*z* from another), these devices allow for us to register tens of thousands of peptides belonging to thousands of different proteins in one experiment. However, the sensitivity of the proteomic method as a whole is more than 50,000 molecules of the analyzed substance.

For example, the area of the Orbitrap Exploris detector is 60–80 square centimeters, and unlike AFM, does not limit the possibilities of the method. The presence of about 1000 biomacromolecules in 10 µL of sample is required for signal detection. Hence, theoretically, a sensitivity of 10^−15^ M can be achieved by mass spectrometry with an analyte volume of 1 µL.

## 3. Experimental Limits of Proteome Analysis

The sensitivity of an analytical method is determined by the minimum amount of a substance that can be determined or detected by this method [30,31]. The amount of detected biomolecules in solution depends on the characteristics of the analytical method—the detection limit of the analytical method (LoD) [32]. Table 1 presents data on the number of molecules in 1 µL of the sample depending on the initial concentrations of the analyte; this number of molecules should be used as a basis for selecting an analytical method according to the LoD parameter.

The end of the 19th century was marked by the emergence and scientific dissemination in biochemistry of a new technology of signal measurement—mass spectrometric analysis. It is possible to simultaneously record hundreds and even thousands of signals, which after bioinformatic processing, can be associated with chemical structures. This new science has been termed “omical” (from omics—the sum of components within a cell [33]), involving the detection of multiple signals simultaneously. It was assumed that the emergence of such technology would dramatically increase the efficiency of digital medical diagnostics, since obtaining a whole spectrum of signals simultaneously is much more efficient than single measurements and can facilitate the comparison of profiles not even of chemical structures themselves, but of signals characteristic of different diseases. Unfortunately, these goals have remained elusive to date. The limited sensitivity of mass spectrometry techniques does not allow for identification of all components present in a sample. All proteins in a sample (proteomic mixture) can be detected if the sensitivity of the method allows it. If the sensitivity of the method is insufficient, proteins are not detected, and it is often erroneously concluded that they are not present in the analyzed sample at all.

Existing limitations also prevent the development of such a field as single-cell proteomics [34]. Although the mass spectrometry method has emerged as a promising option for the label-free multiplex quantification of target proteins, it is not well suited for analyzing the protein content of single cells or a few cells due to inefficient sample preparation and their small numbers for MS analysis. The main remaining problem is the insufficient amount of protein (less than 0.2 ng) per cell [35]. Therefore, the creation of new highly sensitive methods is as relevant to this field of research as it is for the analysis of liquid biological samples. It is expected that the development of new approaches, as well as the increase in the sensitivity of sample preparation, will make it possible to assess the phenotypic characteristics of each cell and their physiological state to estimate the amount of various types of proteins and their post-stranding modifications associated with cancer, which cannot be predicted by genomic/transcriptomic analysis [36].

The article [37] presents the results of an experiment on a model solution of proteins (UPS1, a mixture of 47 highly purified proteins in identical concentrations), allowing us to determine the limits of experimental possibilities of proteome research. In brief, in order to confirm the hypothesis of insufficient sensitivity and limitations in the dynamic range of measurements for proteomic methods in the analysis of complex biological samples, the experiment was set up as described further below. The UPS1 protein hydrolysate solution was prepared at dilutions ranging from 10^−7^ M to 10^−13^ M as model samples:

(1) Pure UPS1 protein hydrolysate solution.
(2) Solution with a constant ratio of UPS1/*E. coli* protein concentrations (UPS1 protein hydrolysate solution against *E. coli* protein hydrolysate, with UPS1 and *E. coli* concentrations decreasing uniformly during the preparation of the dilutions).
(3) Solution with a changing dynamic range in UPS1 protein concentration (the protein concentration of the tryptic lysate of *E. coli* remained constant, while the concentration of UPS1 decreased during the preparation of dilutions). This model most realistically simulates a biological sample.

**Figure 2:**
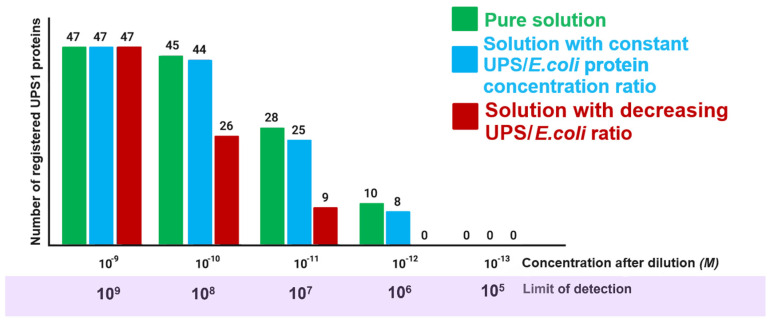
Dependence of the number of proteins recorded by the SRM method on solution concentration (adapted from Vavilov et al. [37]).

Figure 2 presents a histogram showing the dependence of the number of detected proteins on concentration after a series of consecutive dilutions of the initial sample.

When the UPS1 mixture is analyzed in a protein dilution to a concentration of 10^−9^ M, the analytical mass spectrometric method can register all proteins present in the sample (see Figure 2). In this case, the LoD is 10^9^ molecules per 1 µL. If the mixture is diluted to a protein concentration of 10^−13^ M, the method fails to register any protein present at this concentration in the sample (LoD = 10^5^ molecules per 1 µL). This means that the Selected Reaction Monitoring (SRM) method can detect proteins present in the sample at a concentration of at least 10^−12^ M; the limit of detection of this method is 10^6^ molecules per 1 µL (see Figure 2).

However, if we concentrate the proteins present in the concentration of 10^−12^ M to 10^−9^ M, then again, almost all proteins are detected. Consequently, when analyzing proteins in low-concentration solutions, the capabilities of existing technologies do not allow for detecting target objects if the number of molecules present in the sample is below the limit of detection of the method. In our case, it is 10^6^ molecules per 1 µL. The consequence of this experiment is the necessity to specify in any proteomic experiment the LoD of the used method, since the list of registered proteins is not an absolute characteristic of a biological sample, but depends on the type of analytical method chosen; its sensitivity and methodological features, for example, offer a good resolution for a group of proteins possessing certain physicochemical properties.

Probably, a possible reason for the absence of signal from the target protein in low-concentration solutions is the decrease in the “signal/noise” ratio; this value is very small, and sometimes the noise exceeds the signal and the signal from the target biomolecule is lost among the noise. When setting up an experiment, it is necessary to take into account the fact that noise increases with increasing sensitivity, since the sample contains not only proteins, but also other objects that give complexity to the biomaterial and reduce the signal-to-noise ratio. What is fundamental in this situation is that the signal belongs to the protein, hence the signal is localized at the same location in the mass spectrometric profile. When the material is diluted, the signal from the target molecule will remain at the same location, while the noise changes at different locations in the profile [37].

Starting from some point, it is necessary to operate not with the sensitivity parameter of the detector, but with the dilution of the biomaterial in order to increase the ratio of the signal, which has a constant localized site. For this purpose, it is necessary to dilute the biomaterial at the maximum sensitivity of the technology achieved possible and, thus, to try to detect additional protein molecules, which, due to interference of the signal noise with noise, are not manifested at high concentrations of biological material.

Identification of such stable signals amid the noise is one of the possible approaches to overcome the limitations of experimental approaches using bioinformatics methods. By increasing the signal/noise ratio, it will eventually be possible to achieve some dependence of the proteome size (the number of detected proteins) on this ratio and to determine an equation that describes this function.

## 4. Bioinformatic Methods for Analyzing the Complete Proteome of Complex Biological Solutions

The proteome of biological samples contains a significantly larger number of protein species compared to artificial mixtures. It was shown above that currently it is not possible to experimentally detect the entire proteome of a biological object due to limitations of experimental technologies.

To form a complete proteome of human tissues (a list of protein species with indications of the concentration of each species [38]), bioinformatics methods can be used. Based on the experimentally obtained fragment of the proteome, models characterizing the distribution of protein species by concentrations depending on the LoD of the analytical method can be obtained. The models obtained in this way can be used to predict the proteome of the region of the concentration range in which an experimental study is impossible (“semi-bioinformatic proteome”).

Table 2 presents the results of experimental measurements of the proteome of the UPS1 mixture by directional (SRM) and panoramic (Shotgun) mass spectrometric analysis methods. The data were obtained for pure solutions and mixtures with an added matrix *(E. coli* for modeling complex biological systems); the results are represented by the number of detected proteins.

Three peptides for each protein from the UPS1 kit were selected for SRM analysis by the Shotgun approach [37]. As shown in Table 2, at a concentration of 10^−8^ M, the Shotgun approach manages to identify all proteins in the pure UPS1 sample. However, when the concentration is reduced by an order of magnitude, i.e., to 10^−9^ M, there is a sharp decrease in the identified proteins, down to six pieces. This is due to the fact that in this method, ions that exceeded the intensity cutoff threshold will be directed to fragment and register daughter ions. Additionally, ions with intensities that do not exceed the cutoff threshold will not be fragmented, so they will not be assigned peptide sequences from the database during the search algorithm. These limitations do not allow for efficient registration of low-intensity peptide ions of a complex biological sample.

As for SRM for pure UPS1 and UPS1/*E. coli*, the first losses in protein identification begin when diluted to a concentration of 10^−10^ M (Table 2, 45 and 44 proteins, respectively). At a concentration of 10^−13^ M, no more proteins can be identified. The concept of SRM analysis differs from Shotgun in that the researcher must select and explicitly specify the analytes of interest in advance. At the same time, the devices on which this type of analysis is traditionally performed (triple quadrupole) do not have storage elements with limited capacity, so even the weakest signal from an analyte within the device’s capability will be registered [39]. Thus, the analytical sensitivity of the SRM method significantly exceeds the standard Shotgun analysis. However, SRM analysis cannot completely replace Shotgun analysis because of its limited multiplexing capability. In SRM analysis, the highest efficiency is achieved with complete chromatographic separation of all analytes, which is not always easy to achieve.

Based on the experimental data (see Table 2), equations describing the dependence of the number of detected proteins on the sensitivity of the analytical method and protein concentration in the sample were obtained. The initial mixture of UPS1 by a series of serial dilutions was used for the preparation of biological samples with different, known in advance, concentrations of proteins. 

**Figure 3:**
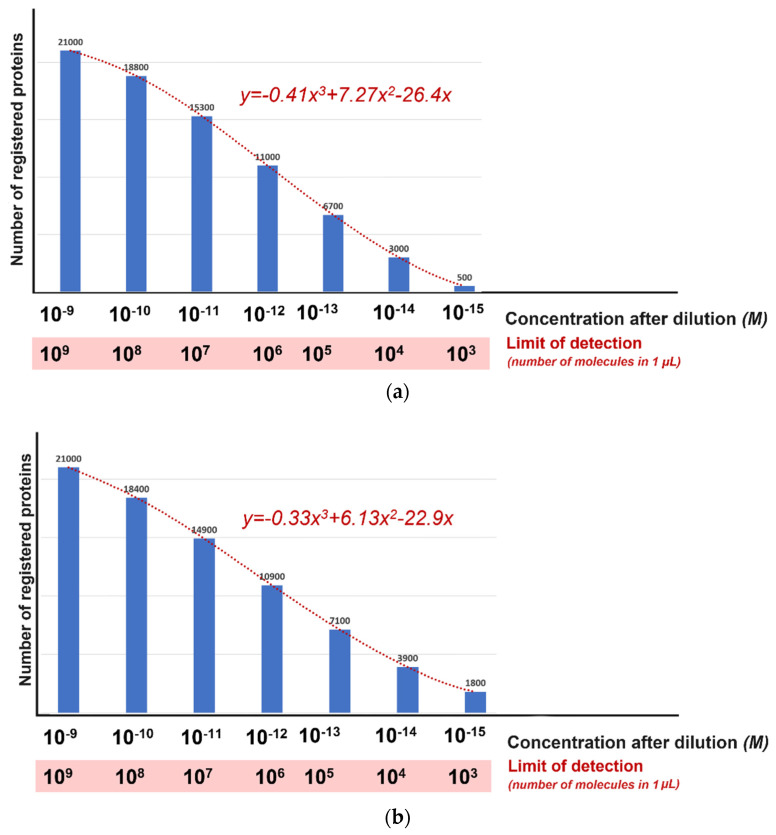
Calculated histograms of the distribution of the number of SRM-registered proteins of the master human proteome (**a**) in pure solution (based on the pure UPS1 model) and (**b**) in biological solution based on UPS1/*E. coli* mixture measurements; see Table 2.

The obtained equations were used to calculate the distribution of the number of proteins that can be registered by the SRM method from the number of proteins forming the master (excluding proteoforms—one gene and one protein [38]) proteome. The calculation was performed in two cases—pure solution analysis (Figure 3a) and the biological sample (Figure 3b, based on the model built from the UPS1/*E. coli* mixture measurement data; see Table 2).

According to the obtained calculations, the LoD of the analytical method at the level of 10^3^ molecules per 1 µL l will allow for registering proteins present in concentrations up to and including 10^−15^ M. The obtained models for pure solution and biological sample look identical and have only insignificant differences (see Figure 3).

Obviously, if proteins are present in the sample in the same concentration and a part of these proteins can be registered and the other part remains “invisible”, it is necessary to take into account an additional characteristic of proteins—differences in the physicochemical properties of molecules; this will allow for calculating, for a particular protein, the probability of being registered.

The current capabilities of analytical methods allow us to register, at best, 30–45% of the protein species potentially present in the sample [40,41]. In our earlier work in “the Human Proteome Project”, we obtained information about 158 and 142 transcripts for HepG2 cell line and liver tissue, respectively; SRM/SIS measurements and Shotgun LC-MS/MS allowed us to detect 91 chromosome 18-encoded proteins in total [40].

The use of bioinformatic models to estimate the distribution of the number of detected proteins from the LoD of the analytical method allows us to estimate the completeness of proteome coverage with sensitivity at the level of thousands of molecules in 1 μL (proteins present at a concentration of 10^−15^ M can be registered). This is three orders of magnitude higher than the experimental capabilities of modern mass spectrometric methods.

## 5. Conclusions

Currently, the complete proteome is not known for any biological entity. Modern proteomics is shortsighted and cannot distinguish between situations when there is no real target protein in the sample, or there is such a protein, but the number of molecules of this protein is insufficient to trigger the detector of the recording device. Is it possible to create methods that allow for registering single molecules similar to the G-M counter, when it is possible to register every molecule within the radius of the counter sensitivity? Basically, yes, but so far, no such technological systems have been produced.

A fundamental idea justifying the necessity of creating a new bioanalysis technology is proposed. A model case of uniform distribution of all the sought molecules throughout the entire volume of a biological sample is considered. For a complete inventory of all proteins, according to calculations, one molecule is required to be detected in 1 μL. Analytical methods with sensitivity better than 10^−15^ M need to be developed. In the first step, the instrumental and technological problem must be solved. In the next step, with the necessary technology, it is possible to determine the complete proteome of the samples and quantify the content of all proteins. Applying this technology to analyze biosamples from healthy volunteers and patients, it is possible to establish the protein profile and its correlation with the state of the organism’s “health” or “disease”. Once the amount of a particular protein corresponding to a certain state of the organism has been determined, it will be possible to create clinical diagnostics. The new technology can become the basis for preventive and predictive medical diagnostics.

In the future, it will be possible to allow for the installation of such a sensor in the blood circulation system, which will register the fact of passing through the sensor target molecule.

Creating new diagnostic systems and implementing them in medical practice is one of the most labor-intensive tasks. For example, the approaches of Theranos Corporation, which promised to revolutionize medical diagnostics, although they were implemented, later became a cause for scandals. Theranos technology implied blood sampling from a finger (several microliters) into a disposable cartridge, which was then loaded into a “reader” for analysis. The advantages were the ability to obtain results quickly, as well as multidisciplinary screening on a single blood sample [42]. It was later learned that Theranos equipment gave inaccurate results for about one in ten tests, resulting in thousands of unnecessary and negative experiences of patients [43]. To avoid similar situations, a lot of fundamental work, verification of the reproducibility of technologies by independent commissions, openness and transparency of the results of scientific research are necessary.

## Figures and Tables

**Table 1:** Number of molecules in 1 µL of blood at different analyte concentrations (adapted from Pleshakova et al. [24]).

Concentration, M.	Number of Molecules in 1 µL (LoD)
10^−6^ (micro)	10^12^ (trillions, tera)
10^−9^ (nano)	10^9^ (billions, giga)
10^−12^ (pico)	10^6^ (millions, mega)
10^−15^ (femto)	10^3^ (thousands, kilos)
10^−18^ (atto)	1 (units, deca)

**Table 2:** Dependence of the number of detected proteins on the concentration at dilution of the initial mixture of UPS1.

Concentration at Dilution (M)	Protein Counts (SRM, Pure UPS1)	Protein Counts (SRM, UPS1/*E. coli*)	Protein Counts (Shotgun MS, Pure UPS1)
10^−8^	47	47	47
10^−9^	47	47	6
10^−10^	45	44	0
10^−11^	28	25	0
10^−12^	10	8	0
10^−13^	0	0	0
